# Social and Non-social Mechanisms of Inequity Aversion in Non-human Animals

**DOI:** 10.3389/fnbeh.2019.00133

**Published:** 2019-06-21

**Authors:** Lina Oberliessen, Tobias Kalenscher

**Affiliations:** Comparative Psychology, Institute of Experimental Psychology, Heinrich Heine University, Düsseldorf, Germany

**Keywords:** inequity aversion, animals, social vs. non-social theories, moderator variables, task design, choice task

## Abstract

Research over the last decades has shown that humans and other animals reveal behavioral and emotional responses to unequal reward distributions between themselves and other conspecifics. However, cross-species findings about the mechanisms underlying such inequity aversion are heterogeneous, and there is an ongoing discussion if inequity aversion represents a truly social phenomenon or if it is driven by non-social aspects of the task. There is not even general consensus whether inequity aversion exists in non-human animals at all. In this review article, we discuss variables that were found to affect inequity averse behavior in animals and examine mechanistic and evolutionary theories of inequity aversion. We review a range of moderator variables and focus especially on the comparison of social vs. non-social explanations of inequity aversion. Particular emphasis is placed on the importance of considering the experimental design when interpreting behavior in inequity aversion tasks: the tasks used to probe inequity aversion are often based on impunity-game-like designs in which animals are faced with unfair reward distributions, and they can choose to accept the unfair offer, or reject it, leaving them with no reward. We compare inequity-averse behavior in such impunity-game-like designs with behavior in less common choice-based designs in which animals actively choose between fair and unfair rewards distributions. This review concludes with a discussion of the different mechanistic explanations of inequity aversion, especially in light of the particular features of the different task designs, and we give suggestions on experimental requirements to understand the “true nature” of inequity aversion.

## The Concept of Inequity Aversion

Other-regarding preferences, i.e., the consideration of the well-being of others when making decisions, are pertinent in human behavior and economic decision making (Fehr and Schmidt, 1999). Such decisions are not solely based on egoistic, materialistic motives, but others’ outcomes are considered as well. Other-regarding preferences have often been studied with economic games (e.g., Yamagishi et al., 2009; Margittai et al., 2015; Strombach et al., 2015). For instance, in the dictator game, participants are asked to split an endowment between themselves and a co-player. Decades of research with the dictator game has shown that people across many cultures and socio-economic groups voluntarily share money and other resources with others (Bolton et al., 1998; Engel, 2011). Another game is the ultimatum game (Güth et al., 1982) in which one player, the proposer, splits a sum of money between herself and another player, the responder. The responder can decide whether to accept or reject the share. If she accepts, both players can keep their share. If she rejects, both players receive nothing. Several thousand replications of the ultimatum game (Güth and Schmidt, 2013) have revealed that the vast majority of responders rejects offers that are perceived unfair, i.e., they forego own-payoffs, to punish unfair proposers. Yet another game is the impunity game (Bolton and Zwick, 1995). In this game, one player, the proposer, can share an endowment between herself and a second player, the responder. The responder can either accept or reject the offer. If she accepts the offer, both players keep their share, if she rejects, the responder receives nothing while the proposer keeps her share. Unfair offers are often rejected by responders (Bolton and Zwick, 1995), thus leaving them empty-handed with no economic consequences for the proposer. Rejections are puzzling at first sight, but are likely fueled by an emotional response to unfairness, revealing that responders derive more disutility from small, but unfair gains than from no gains at all.

Even though such fairness-driven behaviors appear economically unreasonable on the surface because of their costliness (recipients forego rewards or accept costs to punish fairness violators), they are often considered the consequence of so-called inequity aversion (IA), an affective, cognitive and behavioral response to unequal outcomes (Oberliessen et al., 2016). Generally, two forms of IA can be distinguished: (1) aversion against outcome distributions that yield a higher payoff for a partner relative to one’s own payoff, given matched efforts to obtain the payoff (disadvantageous IA); and (2) aversion against outcomes that produce a lower payoff for a partner relative to one’s own payoff (advantageous IA; Oberliessen et al., 2016).

But what is the benefit of costly IA if it does not increase, or even lowers, an organism’s immediate (economic or Darwinian) fitness? IA has been hypothesized to function as a mechanism to ensure the sharing of payoffs and, thus, to enable and maintain long term cooperation with non-kin. It is proposed to serve as an unfairness detector, protecting individuals from exploitation (Brosnan, 2006, 2011; Brosnan and de Waal, 2014). Cooperation allows individuals to achieve goals that they could not achieve alone (e.g., teamwork in humans, or cooperative hunting and cooperative breeding in non-human animals) and offers the possibility to exchange favors over time (direct, indirect and generalized reciprocity; e.g., delousing behavior in monkeys; Stevens and Hauser, 2004; Brosnan and de Waal, 2014).

## Inequity Aversion in Non-human Animals

This explanation already foreshadows, and the examples imply, that IA might not solely occur in humans, but can also be expected in social non-human animal species that engage in cooperative behaviors. Indeed, evidence has accumulated over the last years suggesting that disadvantageous IA exists in various social species. In 2003, Brosnan and de Waal (2003) published a pioneering study testing the response of brown capuchin monkeys to unequal rewards. In this study, two monkeys in adjacent cages could both exchange a token for a food reward with a human experimenter. In the equity condition, both individuals received a piece of cucumber reward for successfully exchanging the token. In the inequity condition, one of the monkeys received a more valuable grape while the other monkey continued to receive the lower valued piece of cucumber for performing the same token exchange task. As a consequence, the disadvantaged monkey refused to exchange the token, or rejected the cucumber reward entirely, tentatively reminiscent of the behavior of human responders in the impunity game (see below for critical discussion). Since this early study, IA was replicated in capuchin monkeys (van Wolkenten et al., 2007; Fletcher, 2008; Takimoto et al., 2010; Takimoto and Fujita, 2011), and reported in macaques (Massen et al., 2012; Hopper et al., 2013), chimpanzees (Brosnan et al., 2005, 2010), cotton top tamarins (Neiworth et al., 2009), dogs (Range et al., 2009, 2012; Brucks et al., 2016; see McGetrick and Range, 2018 for an overview), wolves (Essler et al., 2017), crows (Wascher and Bugnyar, 2013), rabbits (Heidary et al., 2008) and rats (Oberliessen et al., 2016).

However, some studies failed to demonstrate disadvantageous IA in non-human animals, for example in capuchin monkeys (Dubreuil et al., 2006; Roma et al., 2006; Fontenot et al., 2007; Silberberg et al., 2009), chimpanzees, bonobos, orangutans, and gorillas (Bräuer et al., 2006, 2009), cleaner fish (Raihani et al., 2012), keas (Heaney et al., 2017), and dogs (Horowitz, 2012). While the lack of IA in less cooperative species like orangutans (Bräuer et al., 2009; Brosnan et al., 2011) or squirrel monkeys (Talbot et al., 2011; Freeman et al., 2013) might not come unexpected, given the hypothesis that IA is primarily a mechanism for maintaining cooperation, it is hard to explain its absence in cooperative species like capuchin monkeys, dogs, chimpanzees and cleaner fish (see Table 1 for an overview of all studies). Consequently, there is an ongoing, relatively heated debate about the true nature of IA, whether it truly serves to maintain cooperation, and whether it even exists at all in non-human animals.

**Table 1:** Evidence for and against inequity aversion in non-human animal species using different task designs.

Reference	Species	Task type	Disadvantageous IA	Advantageous IA
Brosnan and de Waal (2003)	Capuchin monkeys	Impunity	+	
van Wolkenten et al. (2007)	Capuchin monkeys	Impunity	+	
Fletcher (2008)	Capuchin monkeys	Choice	+	
Takimoto et al. (2010)	Capuchin monkeys	Choice		+
Takimoto and Fujita (2011)	Capuchin monkeys	Choice		+
Dubreuil et al. (2006)	Capuchin monkeys	No task	−	
Roma et al. (2006)	Capuchin monkeys	No task	−	
Fontenot et al. (2007)	Capuchin monkeys	No task	−	
Silberberg et al. (2009)	Capuchin monkeys	Impunity	−	
De Waal et al. (2008)	Capuchin monkeys	Choice		+
Hopper et al. (2013)	Macaques	Impunity	+	
Massen et al. (2012)	Macaques	Impunity	+	
Ballesta and Duhamel (2015)	Macaques	Choice		+
Chang S. W. et al. (2011)	Macaques	Choice		−
Brosnan et al. (2005)	Chimpanzees	Impunity	+	
Brosnan et al. (2010)	Chimpanzees	Impunity	+	
Jensen et al. (2007)	Chimpanzees	Choice + impunity	−	−
Kaiser et al. (2012)	Chimpanzees	Choice + impunity	−	−
Bräuer et al. (2006)	Chimpanzees, bonobos, orangutans, gorillas	No task	−	
Bräuer et al. (2009)	Chimpanzees, bonobos, orangutans, gorillas	Impunity	−	
Horner et al. (2011)	Chimpanzees	Choice		+
Neiworth et al. (2009)	Tamarins	Impunity	+	
Freeman et al. (2013)	Marmosets, owl monkeys, squirrel monkeys	Impunity	−	
Brosnan et al. (2011)	Orangutans	Impunity	−	
Range et al. (2009)	Dogs	Impunity	+	
Range et al. (2012)	Dogs	Impunity	+	
Horowitz (2012)	Dogs	Choice	−	−
Brucks et al. (2016)	Dogs	Impunity	+	
Essler et al. (2017)	Wolves	Impunity	+	
Wascher and Bugnyar (2013)	Crows	Impunity	+	
Heidary et al. (2008)	Rabbits	No task (histopathology)	+	
Oberliessen et al. (2016)	Rats	Choice	+	
Márquez et al. (2015)	Rats	Choice		+
Hernandez-Lallement et al. (2015, 2016)	Rats	Choice		+
Hernandez-Lallement et al. (2016)	Rats	Choice		+
Hernandez-Lallement et al. (2018)	Rats	Choice		+
Raihani et al. (2012)	Cleaner fish	Impunity	−	
Heaney et al. (2017)	Keas	Impunity	−	

*For each species tested on IA, the particular task type is specified. “Impunity” refers to impunity-like tasks (e.g., token exchange tasks) in which pairs of animals are confronted with equal or unequal outcomes, and they can choose to reject rewards and/or refuse further task performance. “Choice” refers to tasks in which an actor animal can actively choose between an equal and an unequal reward distribution. “No task” implies that equal, respectively unequal rewards are offered by an experimenter for free, and the animals can decide to accept or reject these food rewards. A “+” means that the particular authors found evidence for the respective kind of IA, a “−”means that there was no such evidence*.

## One Concept—Many Theories

In this section, we will more closely consider different theories of IA that have been proposed to account for the heterogeneous results. Some of these theories refer to social motives, but others explain previous alleged IA-like behaviors with non-social cognitive mechanisms.

### Social Hypotheses: Maintaining Cooperation vs. Social Disappointment

Brosnan (2006, 2011) posits that fairness preferences, ultimately leading to IA, are advantageous for an organism because, as mentioned above, they serve as a mechanism to ensure the sharing of payoffs and thus, to enable and maintain long term cooperation with non-kin. However, other authors offer different, more mechanistic interpretations of the animals’ behavior in the above-mentioned tasks. The social disappointment hypothesis (Engelmann et al., 2017) suggests that, rather than being sensitive to the relative advantage of the conspecific, animals actually respond to reward expectations triggered by the human experimenter. According to this hypothesis, the actor animal would simply be disappointed by the experimenter because she is not rewarding it as well as well as he could obviously have. Engelmann et al. (2017) tested their hypothesis in an experiment with chimpanzees. They used a two-by-two design in which food was either distributed by an experimenter or a machine and with a partner present or absent. In accordance with their hypothesis, they found that chimpanzees were more likely to reject food when it was distributed by an experimenter compared to a machine. Rejection rates were unaffected by the presence or absence of a partner chimpanzee. Hence, the authors concluded that the refusal of the less preferred food item stemmed from the social disappointment in the experimenter and not from the violation of the animals’ sense of fairness.

However, this conclusion can be debated, too. First, Engelmann et al.’s (2017) result might be species- and context-specific; for instance, while chimpanzees might emotionally respond to violations of reward expectations associated with their human experimenter, other animals, like rodents and birds, might be less sensitive to their experimenter’s behavior. In addition, this hypothesis is, at closer inspection, not very parsimonious, but makes relatively strong assumptions about the animals’ computational capabilities: disappointment by the experimenter’s bad rewarding performance requires the ability to actually realize that the experimenter could have performed better in providing higher quality of rewards. Finally, the social disappointment hypothesis seems more about the source of unfairness sentiments than about the existence of such sentiments *per se*: the hypothesis is perfectly consistent with the idea that the chimpanzees actually felt treated unfairly, it just predicts that they attributed this negative state to the experimenter, and not to the conspecific; hence, the animals would still show a form of IA.

One way to resolve these ambiguities would be to design tasks without experimenter interference, e.g., tasks in which two individuals have to negotiate the distribution of rewards over successive trials (e.g., Brosnan et al., 2006; Melis et al., 2009). Promising approaches on rule observance and conflict resolution have recently been developed for mice (e.g., Choe et al., 2017), but the implications for IA are still elusive. Future research should focus on the development of inter-conspecific negotiation tasks.

### Frustration Hypothesis

Other authors proposed that non-social motives might also explain the animals’ behavior in IA tasks. For example, Roma et al. (2006) suggested that frustration rather than IA might account for some of the findings. They investigated pairs of capuchin monkeys and offered the “model” monkey grape or cucumber while the “witness” monkey always received cucumber. The authors found that the witnesses’ rejections of cucumber were not dependent on whether the model received grape or cucumber, i.e., they found no evidence of behaviorally measurable sensitivity to inequity. However, they also observed that, when cucumber was offered to the model monkeys who were used to grapes, they showed higher rejection rates of cucumber than the witnesses. This finding suggests that previous experience with a more valuable reward (grape) results in a relative devaluation of the less valuable reward, and, hence, its rejection. Thus, rejections might reflect frustration about the poor reward rather than feelings of unfairness. Nevertheless, it should be noted that the experimental setup differed to the one of Brosnan and de Waal (2003) as the animals received the rewards for free, i.e., without an effort requirement or token exchange. This lack of a cost requirement might be crucial because other research has shown that effort seems to be an important moderator of the magnitude of the IA response (van Wolkenten et al., 2007; Wascher and Bugnyar, 2013). This raises the question of whether the lack of any effort requirement in Roma et al.’s (2006) experiment might explain the absence of IA. Nevertheless, this consideration does not entirely disqualify frustration as a potential, non-social moderator of the animals’ rejection behavior in IA tasks.

### Reward Expectation Hypothesis

A related non-social explanation of the rejection of unequal rewards in IA tasks is the reward expectation hypothesis (Bräuer et al., 2006; see also Dubreuil et al., 2006; Neiworth et al., 2009). The hypothesis states that seeing another individual receiving a more valuable reward raises the expectation of receiving the same valuable reward. Deliveries of less valuable rewards thus violate the animal’s reward expectation. By consequence, reward rejections or refusals of task performance could also be caused by failed expectations and negative reward prediction errors, and, hence, cannot with certainty be attributed to IA. A recent human study provided further evidence for the importance of expectations (Vavra et al., 2018). Participants in an ultimatum game were provided with explicit information on what kind of offers to expect by a certain proposer. The authors showed four different distributions, manipulating both the mean and the variance of these expected sets of offers. They found that 50% of the participants systematically changed their behavior as a function of their reward expectations (Vavra et al., 2018). As only the offer expectations differed between conditions, social processes alone cannot explain the changes in behavior corresponding to these offer expectations.

However, this line of reasoning still leaves room for social processes underlying rejection behavior in IA tasks. In standard reinforcement learning, non-human animals derive reward expectations purely from own-experience with past rewards. But in Brosnan and de Waal’s original experiment as well as in follow-up studies, subjects never received the more valuable reward, so any elevated reward expectations based on own-reward history is unlikely. The reward-expectation hypothesis therefore specifically states that own-reward expectations would be influenced by the perception of rewards delivered to others. But the assumption that perceiving rewards delivered to others vicariously elevates own-reward expectations actually require the existence of social comparison processes, and, hence, implies social cognition; this hypothesis, therefore, cannot qualify as a non-social explanation of the variance in rejection behavior in IA tasks.

Yet, it is still possible that the mere presentation of more valuable rewards raised reward expectations beyond vicarious reward tracking. However, van Wolkenten et al. (2007) pointed out that the more valuable reward in the original task by Brosnan and de Waal (2003) and others was equally visible in both the inequity and equity conditions (the experimenter visibly stored the rewards in front of the experimental cages; van Wolkenten et al., 2007). This symmetry in reward presentation means that a putative presentation-effect on reward expectation is insufficient to explain the higher rejection rates in the inequity compared to the equity condition as the animals could see (and thus expect) the more valuable reward in both conditions. Nonetheless, admittedly, it is still possible that the accessibility of the more valuable reward to the conspecific (inequity condition; the reward is merely visible in the equity condition) might affect the level of expectation (see e.g., Brosnan et al., 2010). Consequently, the fact remains that reward expectation, like frustration, might be another plausible, non-social, moderator of IA.

### Reference-Dependent Reward Valuation and Loss Aversion

Chen and Santos (2006) offer yet another non-social mechanism to account for the rejection behavior in all types of IA tasks. They suggest that reference-dependent reward valuation and loss-aversion can account for the evolution of IA. Reference-dependent reward valuation refers to the subjective evaluation of reward magnitude, or reward quality, relative to a benchmark criterion, such as a standard reward; i.e., a given reward magnitude might be valued differently, depending on whether it is higher or lower than the reference reward magnitude (Marsh and Kacelnik, 2002; Chen et al., 2006). Loss-aversion describes the overweighting of negative reward magnitudes during reward evaluation, i.e., reward magnitudes that are lower than expected, or the overweighting of actual losses, respectively (note that losses are difficult to implement in animal research; most research on loss aversion in animals operationalizes losses as negative deviations from a reference point; Chen et al., 2006).

Chen and Santos (2006) maintain that the monkeys’ behavior in the original IA task (e.g., Brosnan and de Waal, 2003) could be explained by translating reference-dependency and loss aversion concepts to the social domain; that is, they assume a socially generated reference point. According to this idea, the payoff to the other individual in Brosnan and de Waal’s (2003) task might become the reference point against which own-rewards are evaluated. Own-rewards below this reference-point, i.e., cucumber instead of grape, would then be perceived as a loss, generating frustration and loss avoidance, and hence rejection (Chen and Santos, 2006).

### Summary

Thus, in summary, there are a number of social explanations for the animals’ rejection patterns in IA tasks, including genuine fairness preferences and social disappointment, but a range of non-social motives have also been proposed to account for the animals’ behavior, including frustration, reward expectation, reference-point dependency and loss aversion. Note that the different social and non-social motives are not necessarily mutually exclusive, but might work in concert to influence behavior in IA tasks. Furthermore, it is worthwhile pointing out that particularly the non-social explanations are conceptually similar. Reward expectation might be considered a direct result of reference-dependent reward valuation, and hence frustration might occur as a result of loss aversion. The two social explanations mainly differ in the causal attribution of IA, as both assume a form of social disappointment: Either in the human experimenter who rewards below his best or in the relative unfairness between subject and partner. Interestingly, the explanation by Brosnan (2006, 2011) can also be seen as a (social) subcategory of reference-dependent reward valuation (the reference point is the outcome of the partner) and, in addition to that, any form of disappointment might eventually result in frustration.

In the next section, we will consider further moderators of IA. We especially highlight the importance of considering the particular characteristics of the different experimental designs used to elicit inequity aversion. We attempt to link these moderator variables, especially the task design, to the abovementioned theories on IA and provide suggestions for future research.

## The Experimental Design and Other Moderators of Inequity Aversion

There are a number of variables that moderate the extent, or even existence, of IA. As already mentioned, effort seems to be an important moderator of the magnitude of the IA response (van Wolkenten et al., 2007; Wascher and Bugnyar, 2013). Furthermore, the quality of the relationship between the pairs of animals tested in an IA task has been shown to influence the level of IA (Brosnan et al., 2005; De Waal et al., 2008; but see Massen et al., 2012; Brosnan et al., 2015). Social hierarchy position also seems to moderate the level of IA, such that higher rank is associated with more pronounced IA (Brosnan et al., 2010; Oberliessen et al., 2016; but see Massen et al., 2012). Further social moderators are sex (Brosnan et al., 2010) and personality (Brosnan et al., 2015): male chimpanzees, more than females, responded to violations of inequity, refusing to complete the interaction with the experimenter when the partner received a better reward (Brosnan et al., 2010). Chimpanzees that were rated higher in the extraversion dimension and lower in the agreeableness dimension were more likely to respond to inequity (Brosnan et al., 2015). In a recent human study, the sensitivity to pain was also identified as a factor to predict the experience of unfairness (the more pain-sensitive, the more experienced unfairness; Wang et al., 2019).

Perhaps the most important influencing factor of IA is the experimental setting in which IA is probed. Almost all of the above-mentioned studies on IA in animals are variants of the original experiment by Brosnan and de Waal (2003) in which pairs of animals are confronted with equal or unequal outcomes, and they can choose to reject rewards and/or refuse further task performance. These tasks strongly resemble the design structure of the impunity game (Bolton and Zwick, 1995) developed for humans (see above) because, in both the animal and human tasks, individuals engage in costly refusals of their own reward with no economic consequence to the conspecific/proposer. Due to their prevalence in the non-human animal literature, the different theories about the cognitive mechanisms underlying non-human IA mostly explain the behavioral particularities in impunity-like tasks. Here, we propose that the use of a different task design might enrich the discussion, and shed light on some of the open questions regarding the true (social or non-social) nature of IA. In particular, we suggest that a different IA paradigm—choice-based IA task designs—might be a promising complement to the existing IA literature as they offer the potential to avoid some of the interpretational caveats mentioned in the preceding section.

### Design of Choice-Based Tasks

**Figure 1:**
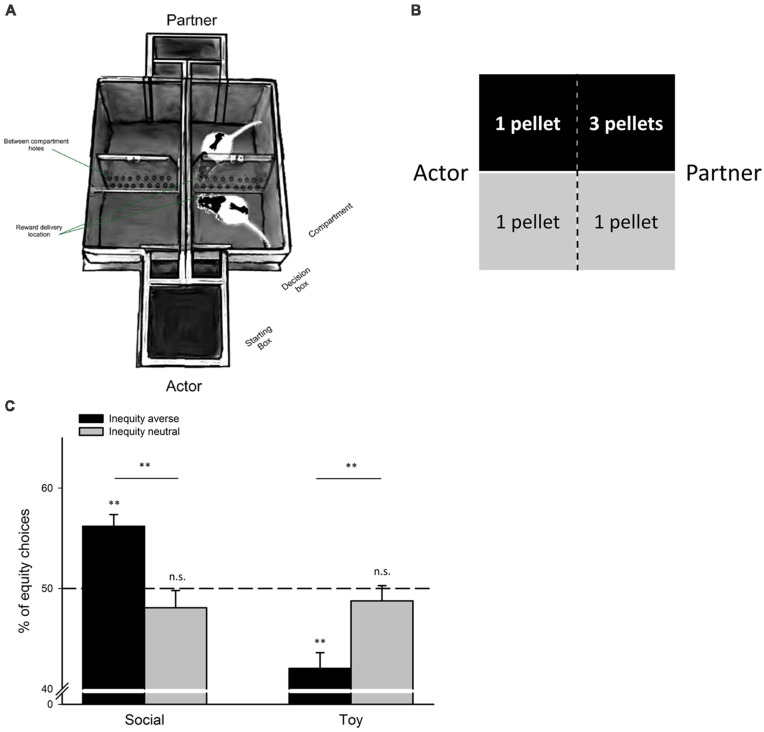
Choice-based disadvantageous inequity aversion task for rats. **(A)** Double T-maze apparatus for quantifying disadvantageous IA in rats. Pairs of rats are trained in this task. The actor rat chooses to enter either an equal-reward compartment, or an unequal-reward compartment. The partner is always directed towards the opposite compartment facing the actor. Actor’s and partner’s compartments are separated by a transparent, perforated wall, allowing rats to see, hear and smell each other, but neither rat can access the other rat’s compartment. The actor rat selects the reward distribution for both rats by entering one of the two compartments in each trial: entering the equal reward compartment produces one food pellet for each rat, entering the unequal-reward compartment yields one food pellet for the actor rat, and three food pellets for the partner rat. Thus, the actor’s decisions are non-costly because its own-payoff is always identical and independent of its choice, but it can choose between a fair outcome (both rats receive the same reward magnitude), or an unfair outcome (the partner rat receives a higher reward than the actor rat). In a non-social control condition (the toy condition), reward contingencies, payoff matrix and all other features of the task are identical, but the partner rat is replaced by an inanimate toy rat. Adapted from Hernandez-Lallement et al. (2015, 2016) with friendly permission by Frontiers in Neuroscience, **(B)** illustration of the payoff matrix, **(C)** rats were classified as inequity averse, or inequity neutral, depending on their individual sensitivity to unequal reward distributions (see Oberliessen et al., 2016 for details). Unlike inequity-neutral rats, inequity-averse rats preferred equal over unequal outcomes in the social, but not in the non-social control condition, the toy condition (**p < 0.01; n.s., not significant). Adapted from Oberliessen et al. (2016) with friendly permission by Elsevier.

In a choice-based task (see Figure 1), an actor animal can actively choose between an equal and an unequal reward distribution, either leaving a conspecific better off (unequal distribution), or equally well off, than the actor animal (equal distribution; see e.g., Fletcher, 2008; Oberliessen et al., 2016). Importantly, the actor animal’s choice is non-costly, i.e., its reward is equal in both reward distributions and thus, independent of the animal’s decision. Preferences for equality are compared between two conditions: a social condition with a conspecific present, and a non-social control condition in which the outcome distributions are identical to the social condition, but the conspecific is absent; e.g., rewards are dropped in an empty, adjacent chamber or compartment. Using such choice-based tasks, it has been shown that both rats (Oberliessen et al., 2016) and capuchin monkeys (Fletcher, 2008) preferred equal over unequal outcome distributions when paired with a conspecific, and that this preference for equal distributions was weaker, or entirely absent, in a non-social control condition with no conspecific present.

In this type of designs, the subject can reveal its fairness preference by its choice, and thus control if inequity occurs at all. The clear advantage of such choice-based IA designs is that the animals do not need to forego own rewards to express their aversion to inequity; thus, they differ from the impunity-like flavor of previous IA tasks that involved costly refusals of own-rewards. This is an important design feature as egoistic desires to maximize food intake in standard impunity-like IA tasks might override any faint, but non-zero IA motives; by consequence, an existent IA preference in an impunity-like task might be masked by an overly strong dislike of sacrificing own-rewards, and it might thus remain undetected.

### The Added Value of Choice-Based Tasks

Choice-based tasks allow to control for some of the alternative factors discussed above that are supposed to influence IA. First of all, because the reward distributions and, hence, rewards to the actor animal, are identical between the social and the non-social condition, frustration effects and violations of reward expectation are unlikely to account for the higher preference for equal-reward outcomes in the social compared to the non-social control condition (but see below for more in-depth discussion of possible further frustration and reward expectation effects in choice-based tasks). Hence, differences in behavior between conditions can more plausibly be attributed to the social component of the task (however, note that many impunity-like IA tasks also had a non-social control condition).

Another reason why fairness-preferences in choice-based tasks cannot easily be explained by frustration effects or violations of reward expectations is the invariance in own-reward value; that is, frustration and reward expectations should only occur if the animal had previous experience with more valuable rewards. However, because own-reward quality and magnitude, as well as delay-to-reward and other reward parameters, are always identical in all trials, irrespective of the actor animal’s choice, the subjects in choice-based tasks have no previous experience with better rewards, making frustration and expectation effects unlikely.

For the same reason, reference-point-dependence and loss-aversion (Chen and Santos, 2006) are also unlikely explanations of equity preferences in choice-based tasks. Because of the invariance in own-reward outcomes, choice-based tasks entail no reference-dependent reward evaluation or negative deviations from a standard reward (i.e., losses).

A counterargument holds that, at closer inspection, some design features of choice-based tasks might actually prompt frustration, reward expectancy and/or reference-dependency effects, albeit in more subtle ways: the total reward magnitude, i.e., the sum of rewards to the actor animal and the conspecific (or empty compartment, respectively), is higher after unfair than fair choices. This difference in total reward magnitude might affect the level of expectation, it might set a reward magnitude standard, and the actor animal might be frustrated because of the inaccessibility of the reward in the other compartment. These reward expectation, reference and frustration motives might bias choice away from the unfair alternative.

However, if these non-social mechanisms indeed favored equity preferences in choice-based IA task, their influence on choice should be stronger in the non-social control than the social condition, for the following reason: in the social condition, the conspecific has access to the reward and consumes it swiftly, but in the non-social condition, the reward is just dropped in an adjacent compartment without being consumed by an (absent) conspecific. Because of the lack of reward consumption in the control condition, the inaccessible reward in the other compartment is displayed longer than in the social condition. This means that the difference in reward magnitude, and, in particular, the inaccessibility of reward, is more salient in the control than the social condition. By consequence, frustration effects and other non-social drivers of preferences should favor equity choices in the control condition more than in the social condition. Yet, this is inconsistent with the choice data, revealing clear preferences for equity choices in the social, but not the non-social condition. Thus, we consider it implausible that non-social aspects of the task explain the condition-effects on equity preferences.

Finally, disappointment in the human experimenter (Engelmann et al., 2017) can be ruled out in choice-based tasks since the experimenter is not responsible for the choice of reward distributions and is present in both the social and the non-social control condition, or he is even entirely absent if tasks are fully automated.

Of course, there might be additional factors that could bias choices towards one or the other alternative in choice-based IA tasks. For example, the actor animal’s perception of the conspecific’s reward consumption might incite reward expectancy or might shift reference points, and the fact that the conspecific consumes a reward that the actor animal cannot access might be perceived as frustrating by the actor. It remains to be determined whether these factors are of social nature (e.g., frustration as a consequence of envy-like emotions about the conspecific’s reward consumption), or non-social nature (e.g., the conspecific’s reward consumption might simply cue the availability of higher rewards that are, however, inaccessible to the actor rat), and it should be investigated if these factors indeed play a role in influencing choice behavior in choice-based IA tasks at all.

### Do Choice-Based Tasks Measure Inequity Aversion?

One crucial question is, whether choice-based tasks actually measure the same thing as impunity-like tasks. That is, is a rejection of an unfair offer in an impunity-like task driven by the same mental and affective mechanisms as preference for equity outcomes in a choice-based task, or are the animals’ decisions in the respective tasks qualitatively different? Rejections of unfair offers in impunity-like tasks clearly have an affective flavor, while preferences for equal outcomes in choice-based tasks do not necessarily reveal strong emotions. However, empirical evidence that impunity-like tasks involve stronger negative emotions than choice-based tasks is elusive; hence, putative differences in the affective domain between task designs are somewhat speculative.

The answer to the question whether impunity-like or choice-based tasks measure the same form of IA also depends on the particular definition of IA used. Fehr and Schmidt (1999), who developed a theory of IA for human decision-makers, defined inequity aversion as the resistance against inequitable outcomes. They stressed that the aversion against inequity can, but does not have to, go along with the willingness to forego material payoffs for the sake of fairness.

It is also conceivable that IA is a special form of temporal discounting (Stevens and Hauser, 2004; for an overview of temporal discounting see Kalenscher and Pennartz, 2008): IA might be the rejection of a sooner smaller reward (an unequal small payoff) compared to a more valuable reward in the future (fair, high rewards in a successful long-term cooperation).

Both definitions of IA entail the willingness of the decision-maker to incur costs for the sake of equity. Since decisions in the impunity-like designs of IA are costly, but decisions in choice-based tasks are not necessarily costly, the construct measured in the former class of tasks comes closer to the definition of IA as put forward by Fehr and Schmidt (1999) or the idea of temporal discounting. Future research should manipulate the costs of the fair option in choice-based designs, and investigate whether animals are also willing to forego own-payoff for the sake of equitable outcomes in these tasks.

In conclusion, we argue that the use of choice-based IA tasks may shed light on some of the remaining open questions raised by experiments using impunity-like IA tasks. We want to stress that we do not consider choice-based IA tasks superior to impunity-like tasks; they merely complement the existing research. We maintain that the combination of both tasks should be the way forward in future research.

## Advantageous Inequity Aversion

This review focused primarily on moderators and mechanisms of disadvantageous IA, and its putative ultimate reasons. The motivation for prioritizing the coverage of disadvantageous over advantageous IA, the aversion against outcomes that produce a lower payoff for a partner relative to one’s own payoff, is that advantageous IA is rarely found (and tested) in impunity-like tasks (Jensen et al., 2007; Horowitz, 2012; Kaiser et al., 2012). However, there are several choice-based IA tasks prompting advantageous IA (also labeled as prosociality or mutual-reward preferences) in different non-human animals, e.g., rats (Hernandez-Lallement et al., 2015, 2016, 2018; Márquez et al., 2015), capuchin monkeys (De Waal et al., 2008; Takimoto et al., 2010; Takimoto and Fujita, 2011), chimpanzees (Horner et al., 2011), and rhesus macaques (Ballesta and Duhamel, 2015; but see Chang S. W. et al., 2011). Similar to disadvantageous IA, the expression of the animals’ aversion against advantageous inequity in choice-based tasks is not costly: the own-reward to the deciding animal is always identical and independent of the choice of a fair or unfair alternative. To date, it is unclear if a principle mental component underlies preferences for equal reward distributions in disadvantageous and advantageous IA settings in non-human animals.

This review mainly focuses on IA in non-human animals. It is important to note that IA has been extensively studied in humans, too, with a vast, partly diverging literature in several different disciplines, including economics and psychology. The terminology and experimental methodology used and covered in this review are largely consistent with the literature in economics, where advantageous IA is defined as preference for fair vs. unfair outcomes, and where IA is mainly investigated by means of economic games (e.g., Fehr and Schmidt, 1999). By contrast, psychologists often label advantageous IA *guilt* and frequently focus on self-reports which can be linked to behavioral intentions underlying other-regarding preferences (e.g., Schmitt et al., 2000), and related concepts, like, e.g., *morality*, *justice*, or *ethics*. We argue that studying IA in animals is not only interesting by itself, but paves the way for harmonizing semantic differences between disciplines as well as highlighting conceptual similarities.

## Neural Substrates of IA

Parallel to behavioral studies on IA, another field of research evolved with the technical progress of cognitive neurosciences. Modern neuroimaging methods offer more and more possibilities to directly study brain processes during social decision making (mainly in humans), and thus to learn more about the underlying mechanisms and brain structures. Although this should not be the focus of this review, we consider it worthwhile to shortly touch on this topic and present some interesting results (note that we do not claim to provide a comprehensive overview; for more details, see Ruff and Fehr, 2014). Several studies which investigated neural responses to disadvantageous and advantageous IA in humans suggest that the dorsolateral prefrontal cortex seems to be particularly involved in encoding and interpreting payoff inequalities and implementing inequality averse behaviors (Sanfey et al., 2003; Hsu et al., 2005; Haruno and Frith, 2010; Tricomi et al., 2010; Chang L. J. et al., 2011; Fliessbach et al., 2012; Cappelen et al., 2014; Güroğlu et al., 2014; Haruno et al., 2014; Yu et al., 2014; Nihonsugi et al., 2015; Holper et al., 2018). Tricomi et al. (2010) found that inequality averse preferences were also correlated with activity in the valuation network (Bartra et al., 2013), mainly ventral striatum and ventromedial prefrontal cortex in humans, suggesting that own-reward activity in the valuation system was modulated by the degree of inequality relative to a better or worse reward received by another participant. A recent study by Gao et al. (2018) even distinguished between neural correlates of advantageous vs. disadvantageous IA. They found that the processing of advantageous inequity involved the left anterior insula, the right dorsolateral prefrontal cortex, and the dorsomedial prefrontal cortex. Disadvantageous inequity correlated with activity in the left posterior insula, the right amygdala, and the dorsal anterior cingulate cortex.

In the animal domain, a study on rhesus monkeys provided evidence that striatal neurons play a role in identifying the social actor and own reward in a social setting (Báez-Mendoza et al., 2013), consistent with the human evidence presented by Tricomi et al. (2010). As mentioned above, the amygdala also seems to play an important role in social decision making (Gao et al., 2018). In line with amygdala’s hypothesized role in social cognition, Chang et al. (2015) could show that basolateral amygdala neurons signaled social preferences in rhesus macaques and mirrored the value of rewards delivered to self and others when monkeys were free to choose. In line with this finding, Hernandez-Lallement et al. (2016) found that basolateral amygdala lesions abolished mutual reward preferences in rats.

Thus, in summary, evidence from cognitive neuroscience suggests that the brain’s valuation system, including ventromedial prefrontal cortex and ventral striatum, as well as a range of structures involved in planning and cognition (dorsolateral prefrontal cortex), emotional processing (amygdala) and the appraisal of negative events (insula) are involved in processing IA in humans as well as non-human animals.

## Conclusions

The main purpose of this review is to highlight some of the open questions and, especially, locate potentially essential differences in the various task designs used to probe IA in non-human animals. Future studies should investigate how animals perform in both impunity-like and choice-based variants of disadvantageous IA tasks to learn about the effect of design-specific differences on IA expression, and to test whether the level of IA in the choice-based task can predict the probability to reject rewards in the impunity-like task, or vice versa. Thus, identifying the commonalities and differences in behavior between both types of tasks will help to better differentiate between theories of IA, and to better understand the actual mental mechanisms underlying IA. Furthermore, future research should compare preferences for fair outcomes in disadvantageous IA tasks with preferences for fairness in advantageous IA tasks with the same individuals. This would help to untangle whether both forms of IA are positively or negatively correlated (respectively correlated at all). It is possible that highly disadvantageously inequity averse individuals do also show higher scores of advantageous IA. On the other hand, it is also conceivable that a high sensitivity of being disadvantaged goes along with a reduced sensitivity towards others being disadvantaged. The clarification of this issue might be further supported by additional neuroscientific studies. Isolating the differences, commonalities, moderators and predictors of each type of IA will yield important insights into the mechanistic underpinnings of IA.

## Author Contributions

LO developed the first concept of the article, wrote the article and revised the article. TK revised the concept of the article, wrote the article, and revised the article.

## Conflict of Interest Statement

The authors declare that the research was conducted in the absence of any commercial or financial relationships that could be construed as a potential conflict of interest.
